# Shear-induced chemical segregation in a Fe-based bulk metallic glass at room temperature

**DOI:** 10.1038/s41598-021-92907-4

**Published:** 2021-07-01

**Authors:** D. V. Louzguine-Luzgin, A. S. Trifonov, Yu. P. Ivanov, A. K. A. Lu, A. V. Lubenchenko, A. L. Greer

**Affiliations:** 1 WPI Advanced Institute for Materials Research, Tohoku University, Sendai, 980-8577 Japan; 2 Mathematics for Advanced Materials-OIL, National Institute of Advanced Industrial Science and Technology (AIST), Sendai, 980-8577 Japan; 3 National University of Science and Technology “MISiS”, Moscow, 119049 Russia; 4 Physics Faculty, Lomonosov Moscow State University, Moscow, 119991 Russia; 5 Department of Materials Science and Metallurgy, University of Cambridge, Cambridge, CB3 0FS UK; 6 School of Natural Sciences, Far Eastern Federal University, Vladivostok, 690950 Russia; 7 Department of General Physics and Nuclear Fusion, National Research University “Moscow Power Engineering Institute”, Moscow, 111250 Russia

**Keywords:** Materials science, Physics

## Abstract

Shear-induced segregation, by particle size, is known in the flow of colloids and granular media, but is unexpected at the atomic level in the deformation of solid materials, especially at room temperature. In nanoscale wear tests of an Fe-based bulk metallic glass at room temperature, without significant surface heating, we find that intense shear localization under a scanned indenter tip can induce strong segregation of a dilute large-atom solute (Y) to planar regions that then crystallize as a Y-rich solid solution. There is stiffening of the material, and the underlying chemical and structural effects are characterized by transmission electron microscopy. The key influence of the soft Fe–Y interatomic interaction is investigated by ab-initio calculation. The driving force for the induced segregation, and its mechanisms, are considered by comparison with effects in other sheared media.

## Introduction

Shear deformation can induce separation of particles of different sizes in colloids and granular media^1,2^. Such behavior is, however, unexpected in the deformation of solid engineering materials consisting of chemical elements with strong chemical bonding, especially when the shear is at room temperature and under mild wear conditions. Here we report observations of shear-induced chemical separation inside an engineering material, namely a metallic glass at room temperature. Metallic glasses (MGs) exhibit a unique combination of mechanical properties^3^: high hardness, high yield strength, large elastic deformation^4^ and high fracture toughness^5^. They have high macroscopic^6,7^, micro-^8^ and nano-scale^9,10^ wear resistance, which is further improved by surface oxides^11,12^. Owing to their high wear resistance^9,13^, good surface quality^14^ and absence of granular structure, MGs are applicable in micro-electro-mechanical devices^15,16^ in which the mechanical contact area between the component surfaces ranges from micrometer to sub-micrometer scale. In MGs plastic deformation is sharply localized in shear bands, and there are clear indications that this can induce chemical segregation within the shear bands^17^. Possible shear-driven segregation is explored further in the present work.

Fe-based alloys form some of the strongest and most wear-resistant MGs^4^, and they can be formed in bulk (i.e. with minimum dimension exceeding 1 mm). Though the glass-forming ability of, for example, Fe–Cr–Mo–C–B alloys^18^, is not too high it can be significantly improved by the addition of rare-earth (RE) metals such as Y^19,20^ and Tm^21^. The combination of relatively good glass-forming ability, high strength^18^ and wear resistance^22,23^ makes a Fe_48_Cr_15_Mo_14_C_15_B_6_Y_2_ bulk metallic glass (BMG) very attractive for micro-gears, while the internal structural changes resulting from nanoscale wear are of fundamental interest. The present manuscript discusses astonishing structural changes observed on wear testing of this material. These changes are connected with shear-induced chemical separation inside the MG, leading to a new mechanism of local deformation stiffening.

## Results

### Initial structure

The as-prepared BMG sample characterized by TEM was found to be glassy (Supplementary Fig. S1). The surface of non-noble metallic samples is expected to be oxidized. XPS applied to characterize and determine the oxide depth profiles with sub-monolayer accuracy (Supplementary Fig. S2) indicated existence of a 1.7 ± 0.3 nm thick oxide layer containing all four metals (Supplementary Table S1).

### Wear testing

The Fe_48_Cr_15_Mo_14_C_15_B_6_Y_2_ BMG is found to have an exceptionally high hardness HV of ~ 12 GPa. The wear tests were performed in AFM with a diamond tip of radius ~ 25 nm (Supplementary Fig. S3a,b). The effective tip radius increases from 25 to 50 nm after 10 passes by attachment of wear products.

**Figure 1:**
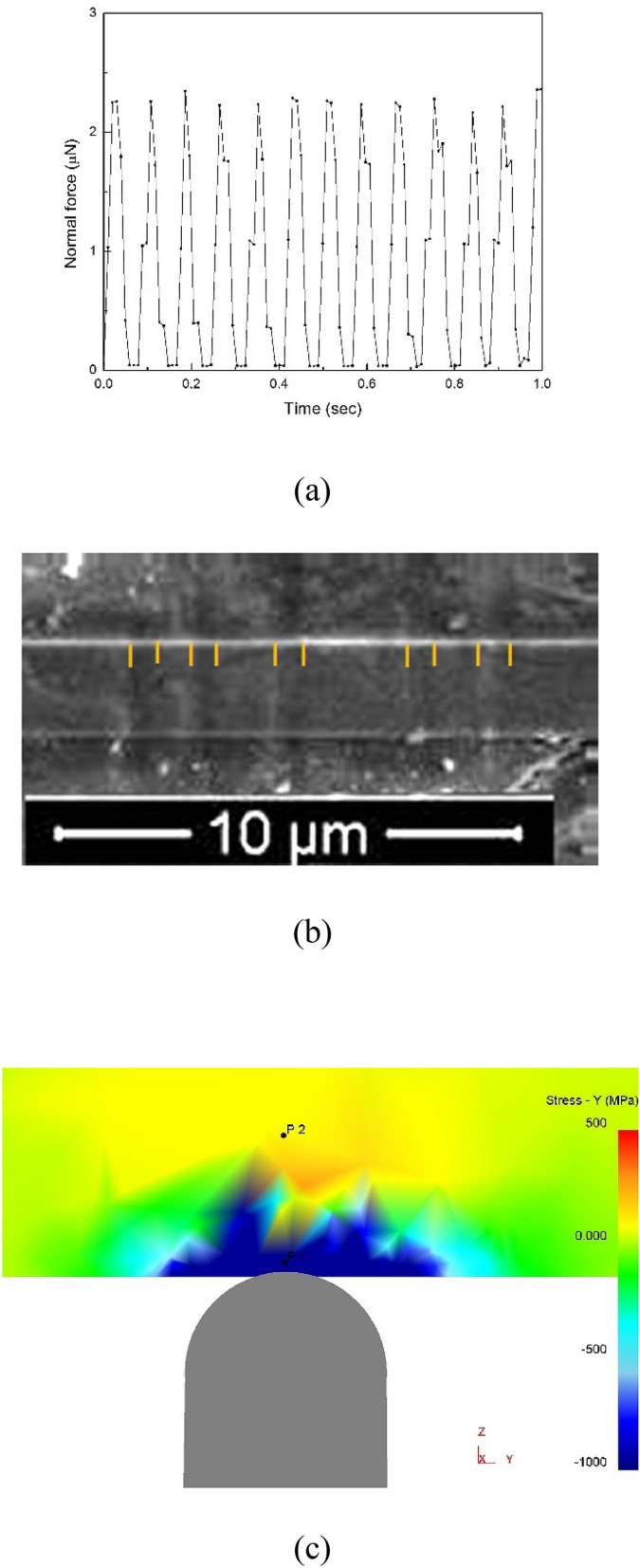
(**a**) Oscillation of normal force in AFM recorded by an oscilloscope during AFM wear test. (**b**) SEM image showing five similar wear tracks on the surface of the Fe_48_Cr_15_Mo_14_C_15_B_6_Y_2_ BMG (the marks indicate the position of the edge of each track; the rectangular patch on top of the tracks is the Pt protective layer deposited by electron beam for the FIB processing). (**c**) Map of the biaxial stress value in X–Y plane (parallel to the sample surface) calculated by FEM. Tensile stresses are marked in red and yellow, compressive stresses in green and blue.

Contrary to other non-ferrous BMGs studied earlier^11^ the studied Fe-based BMG confirmed its expected high macroscopic wear resistance when no plastic deformation of the BMG was detected after several runs in the constant-load mode and after a single run in the oscillating load mode (Fig. 1a). Ten to-and-fro scans with the oscillating load were required to obtain a wear profile. Scanning electron microscopy (Fig. 1b) shows the width of each wear track to be 650‒750 nm. This width indicates that the ten scans in each track follow parallel, but not exactly coincident, lines. On BMG samples with smoother surfaces, such multiple scans give overall wear tracks that are much narrower.

The significant material loss after 10 runs is attributed to delaminating wear (where the wear products are metallic flakes) of the sample^24^ facilitated by the oscillating-load mode of the AFM needle (Fig. 1a). The stress distribution inside the BMG sample loaded with a diamond tip of 25 nm radius was modeled in three dimensions with finite-element modeling (FEM) software DEFORM. The computation details are given in Supplementary Information. As one can see in Fig. 1c, the maximum compressive stress in the X–Y plane is at P1 which is 5 nm from the surface, while maximum biaxial tensile stress of 350 MPa is found at about 30 nm from the surface (P2). Biaxial tensile stresses of lower magnitude persist inside the simulated body to 50 nm depth and more.

AFM also allowed measurement of Young’s modulus *E* of the material. In the wear track, the measured *E* is higher than in the surrounding undeformed area. This apparent stiffening of the sample as a result of deformation is opposite to the decrease in *E* typically found for BMGs (for example, see data for a Zr-based BMG tested under similar conditions, Supplementary Fig. S4). When the wear test was performed at constant load of 1 μN, no increase in *E* was found in the wear track (Supplementary Fig. S3c,d), which is as expected for BMGs that show shear softening.

Wear conditions and possible temperature rise were analyzed using wear maps^25^. Calculations (see Supplementary Information S2) show that the temperature rise on wear is safely negligible.

### Induced structural changes

**Figure 2:**
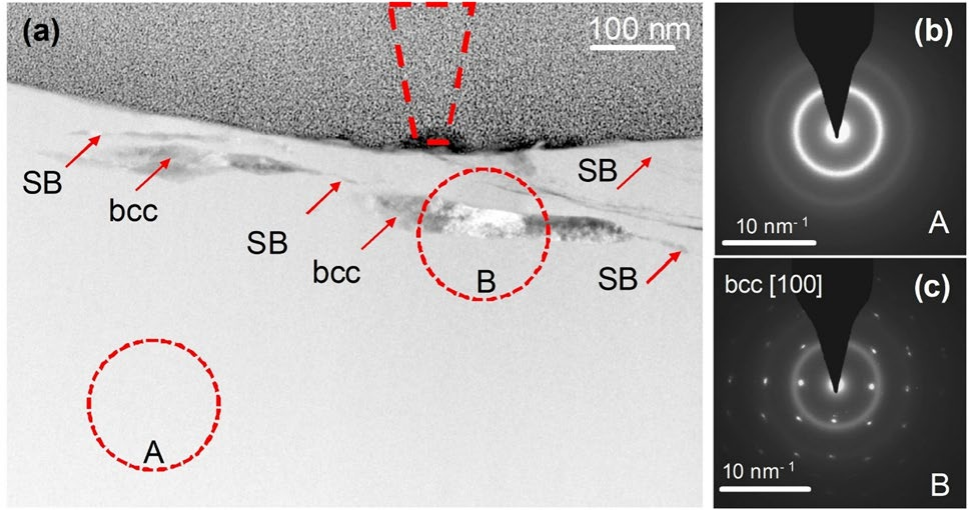
Cross-sectional TEM observation of a wear track. (**a**) HAADF STEM image of the cross-section of the track after ten to-and-fro passes, showing the MG matrix and within it shear bands (SBs) and bcc crystals. The position and size of the AFM tip is indicated by the dashed cone located at the center of the track, which overall is 650‒750 nm wide. (**b**) and (**c**) are SAED patterns from regions A and B, respectively, indicating a glassy phase and a crystal induced by mechanical deformation. The A and B circles on the image show the size of the selected-area aperture used for SAED acquisition.

**Figure 3:**
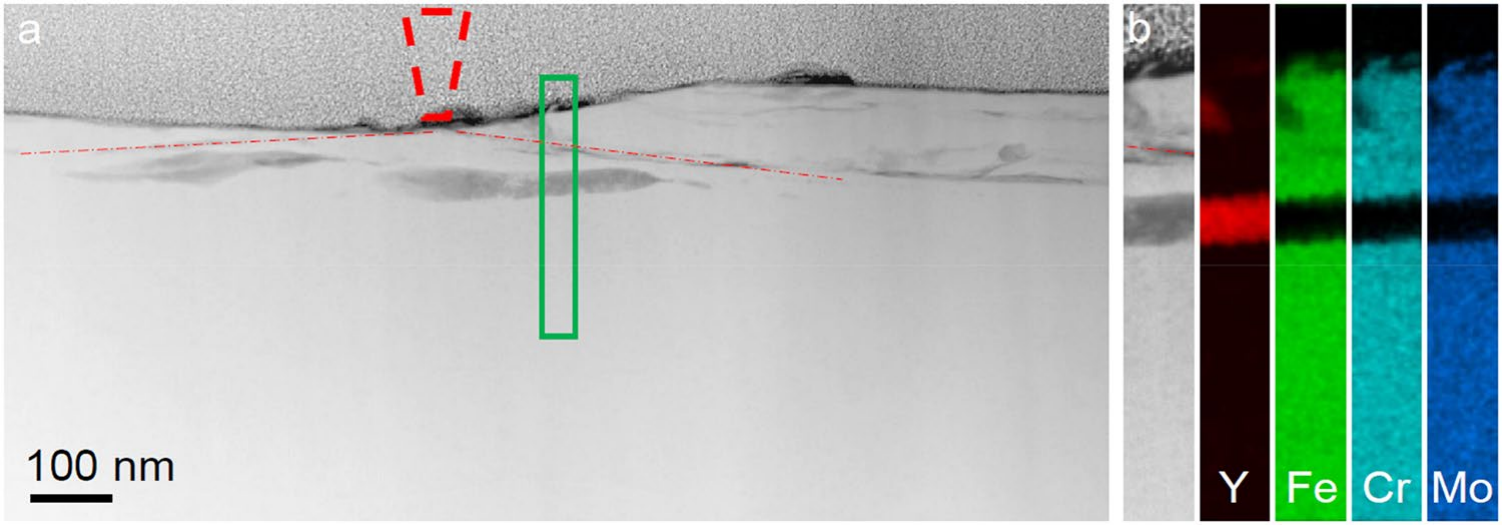
Compositional changes in the material underneath the wear track. (**a**) HAADF STEM image of the cross-section of the wear track shown in Fig. 2a. Similarly, the position of the AFM tip at the center of the track is shown by the dashed cone. The red dashed-dotted lines indicate the position of the shear bands generated during the wear test. (**b**) HAADF image and EDX elemental mapping of the region marked by the green rectangle in (**a**). The EDX maps show that there is no significant difference in composition at the location of these shear bands; the darker HAADF contrast is due to local reduction in density. There is, however, a large difference in chemical composition between the glassy matrix and bcc nanocrystals.

Strain-softening within shear bands is typical for bulk metallic glasses^26,27^; accordingly, the stiffening observed after the wear testing in a oscillating load mode argues for distinct and significant structural changes in the deformed material. The structural changes underneath the wear track are characterized by transmission electron microscopy (TEM). A thin lamella was cut to show the wear track cross-section. Figure 2a shows the HAADF image of the cross-section of the central wear track seen in Fig. 1b, the upper grey part of the image above the surface of the BMG is the Pt protective layer. A projection of a truncated cone is shown with red dashed lines, and represents to-scale the diamond tip in the center of the wear track, which extends over a width consistent with the 650‒750 nm range in SEM (Fig. 1b). Selected-area electron diffraction (SAED, Fig. 2b) shows the matrix to be glassy. Features interpreted to be shear bands (SBs) formed in the matrix are indicated with red arrows. Plates, appearing 10‒50 nm across in projection, with light and dark diffraction contrast (Fig. 2a), and corresponding SAED (Fig. 2c) are nanocrystals of an A2 bcc phase. From the structure, which is confirmed by the corresponding nano-diffraction maps acquired in the scanning-electron-diffraction mode, and from EDX mapping (Fig. 3a), this phase is identified as the high-temperature bcc polymorph of pure yttrium. The true thickness of these Y layers is difficult to estimate as they are not parallel to the original sample surface. They are ~ 300 nm wide in the image in Fig. 2, and may easily span the full depth of the thin foil (up to 50 nm in this near-surface region). The line profile of yttrium content in the EDX mapping in Fig. 3 suggests that the actual thickness of the crystal plate is ~ 20 nm. Apparently thicker crystalline regions in Fig. 2a most likely correspond to overlapping crystals formed in the adjacent shear bands. As indicated on Fig. 2, the distance between the shear bands can be as little as 20‒30 nm.

The shear bands and Y layers appear under the full width of the wear track. In contrast, no crystallization was found after deformation of a Zr–Cu–Fe–Al BMG (Supplementary Fig. S5).

We attribute increased Young’s modulus in the wear track to the formation of these nanocrystalline layers of bcc phase, as the crystals forming in a metallic glass, in general, possess higher *E* values than the glass. Crystallization of the Fe_48_Cr_15_Mo_14_C_15_B_6_Y_2_ BMG has been studied on heating: it crystallizes by growth on pre-existing nuclei of an intermetallic χ-phase, apparently without any nucleation barrier^28^. In the present case, however, there should be no significant heating during wear (see Supplementary Information S2.2), and no crystalline phases other than bcc Y are detected by TEM (Fig. 3). For elements (Ti, Zr) that show bcc/hcp polymorphism, it is characteristic that alloying with (Cr, Fe, Mo) stabilizes the bcc polymorph. Forcing these solutes into yttrium is expected also to favor the bcc structure.

Taking the measured lattice parameter of 411 pm at 1761 K^29^, and an estimated coefficient of thermal expansion of (9‒15) × 10^‒6^ K^‒1^, we can extrapolate to room temperature to obtain a value of 404 pm. Our measured lattice parameter of the bcc phase of 367 pm (Fig. 2c) is significantly (9%) smaller. Energy-dispersive X-ray (EDX) analysis (Fig. 3) indicates 8 at.% Fe, 3 at.% Cr and 5 at.% Mo dissolved in the bcc Y. As the atoms of these elements are significantly smaller than Y, their presence in a substitutional solid solution would reduce the lattice parameter^30^.

### First-principles atomistic simulations

The bcc polymorph of yttrium should not be stable at room temperature. We investigate this phase using ab-initio simulation, which shows that the lattice parameter of the Y-based bcc solid solution containing 16 at.% of metallic solute elements is 380 pm at 0 K (see Supplementary Fig. S6 for details). Taking into account the coefficient of thermal expansion over the relevant temperature range, this implies a lattice parameter of no more than 381 pm at room temperature, broadly matching the experimental finding (367 nm). The comparison of the observed and simulated lattice parameters cannot be fully quantitative because of the small cell size used in the simulation. Nevertheless, we can conclude that the obtained lattice parameter is reasonable for a bcc-Y phase with the measured solute contents.

## Discussion

### Formation of bcc-Y layers

The TEM results and the shape of the crystals suggest that they are formed by concentration of yttrium atoms on shearing planes under the wear track. This shear-induced crystallization in the glassy phase is associated with an astonishing degree of enrichment, as their yttrium mole fraction among the metallic elements rises by a factor of > 30 compared to the initial BMG matrix. When the yttrium content is so high, it is not surprising that there is crystallization. Although the solute elements are insoluble in any allotropic form of yttrium (either hcp or bcc), and Cr and Mo even do not form intermetallic compounds with it, they remain in solid solution presumably because the formation of the crystalline layers is dominated by mechanical effects and not by thermal diffusion (which must anyway be insignificant at room temperature). We note that such a metastable supersaturated bcc Y solid solution has been found to form, in preference to intermetallic compounds, in Y–Fe alloys on rapid solidification^31^ and even on annealing of a Y–Fe glassy phase^32^.

The Fe–Y system has a negative mixing enthalpy in the liquid up to 8 kJ mol^‒1^ and shows many Fe-rich intermetallic compounds, but no crystalline compounds with Y content higher than in Fe_2_Y^33^. Crystallization of an intermetallic compound at room temperature is unfavorable owing to the high stability of glasses with large solute content while those with low solute content are generally less stable. Of the constituent elements of the BMG in this study, Y has the largest atomic radius. As expected, the diffusion coefficient of Y in bcc Fe and in Fe-based metallic glasses is lower than those of other constituent elements^34^. It is then critical to explain why and how Y with a very low diffusion coefficient in Fe can show concentration into thin layers at room temperature, especially when the original glassy matrix contains only 2 at.% of Y. We suggest that this concentration of yttrium from the Fe-based BMG matrix requires mechano-chemical arguments^35^ to be explained.

As seen in Figs. 2 and 3, the crystalline yttrium layers form in the deformed zone underneath the wear track. Indentation is known to create shear bands^36^. Excess volume is generated in and near to shear bands, as shown by MD simulation^37,38^ and confirmed by direct^39,40^ and indirect^41,42^ experiments. Atomic diffusivities within shear bands are several orders of magnitude higher than in the undeformed MG^43^. Around a shear band there is a region of reduced hardness up to 150 µm thick^26^. It is suggested that during shear there is increased atomic mobility within a region up to 200 nm thick^44^. During deformation, the shear rate is highest in the central plane, diminishing to zero when moving into the rigid material on either side. As the actual bands are thin (as little as 10‒20 nm), the shear rate at the center is extremely high^45^, and it is of interest to consider whether this could drive chemical segregation.

### Analogy with colloidal and granular systems

In colloidal systems, the particles flowing along a channel migrate towards the central axis, where the volume fraction occupied by colloid particles rises detectably^2^; this effect is relevant even in such areas as blood flow and drug delivery^1^. The migration towards the center arises from the gradient in the inter-particle collision frequency inevitable when there is a gradient in shear rate^1^. When there is a mixture of particles of two different sizes, the migration drives segregation by size: segregation of small particles to the center is favored by a high volume fraction of the small particles and a low volume fraction of the large particles, and vice versa^2^. Similar segregation effects are seen in granular flow along a chute: for high overall volume fraction of solid, the small particles in a two-size mixture migrate towards the center^46,47^.

We explore whether such shear-driven size segregation could apply for the atoms in a MG. The extrapolation of a mechanism from the scale of macroscopic particles down to atoms is extreme, but we note that a granular medium (sand) has already been considered as an analog for MGs when considering shear bands^48^, and that structural rearrangements during flow have many similarities in colloidal and metallic glasses^49^. Another example of such an extrapolation is the common application of the Stokes–Einstein relation to link the viscosity and atomic diffusivity in metallic liquids^50^.

**Figure 4:**
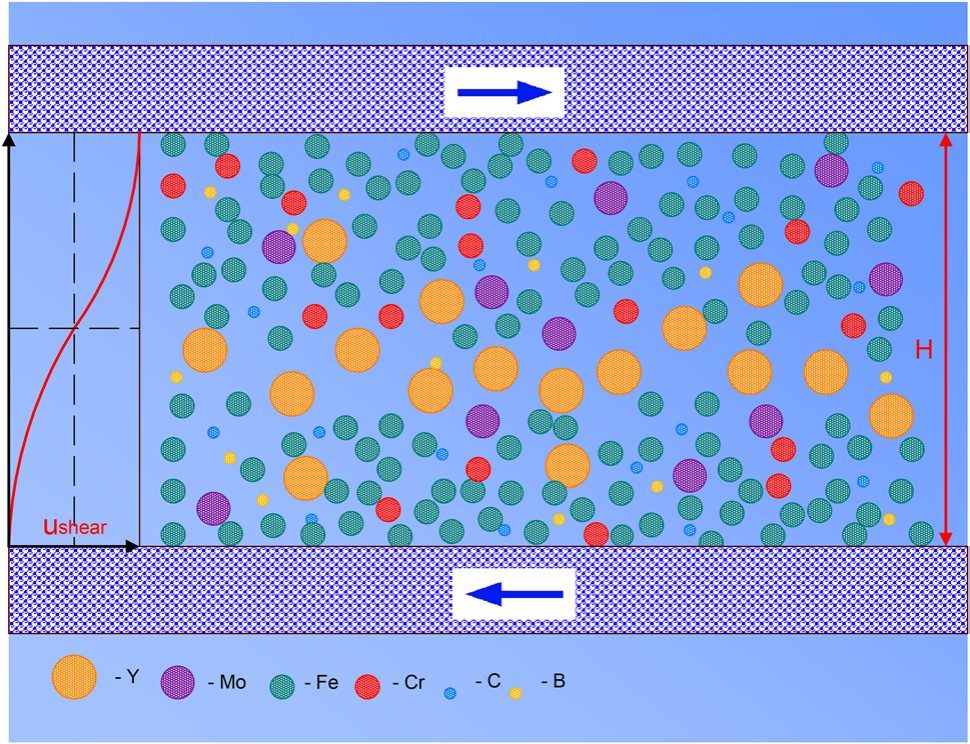
Representation of atomic size-separation in a shear band in a metallic glass. The plot on the left indicates the continuum velocity *u* in the direction of the shear indicated by arrows. From one side of the shear band to the other (i.e. across the full width *H*), the relative velocity is *u*_shear_. A schematic representation of a shear band in which the different atomic species are shown scaled roughly with their Goldschmidt diameters. These diameters are shown roughly eight times larger than would be scaled with a minimum likely *H* of 20 nm, and the depicted packing efficiency is much lower than actual. The largest species (Y) in the MG occupies 2 at.%, which corresponds to ~ 6 vol.% of the MG, and this schematic shows that this species (if behaving as in a colloidal or granular system) is expected to migrate towards the central plane of the shear band, or at least stay in place as the smaller species migrate away.

For axisymmetric flow of a fluid along a flat channel, the velocity of the fluid is zero in contact with the walls and maximum on the central plane (with a parabolic profile across the channel thickness). The velocity gradient, i.e. the shear rate, is maximum at the wall and zero in the center. In a shear band in a solid, the velocity profile, shown in Fig. 4, is inverted in the sense that the shear rate is maximum on the central plane and decays to zero in the rigid material on either side. Thus, segregation towards the central plane in a thin channel would be away from the central plane of a shear band. For fluid flow in a channel of thickness *H*, the characteristic shear rate is *γ′* = 2*u*_flow_/*H*, where *u*_flow_ is the maximum flow velocity. For a shear band of thickness *H*, *γ′* = *u*_shear_/*H*, where *u*_shear_ is the velocity of the solid on one side of the band relative to the other.

Migration of particles is driven by the gradient in *γ′*, which scales as *γ′*/*H*. For monodisperse systems, the migration velocity *u*_mig_ is proportional to *a*^2^, the square of the particle diameter^1^, and we use this scaling to compare a colloidal^2^ and the MG system in the present work. The rate at which segregation becomes apparent is proportional to *u*_mig_/*H*, and thus scales with the parameter (*γ′a*^2^/*H*). Table 1 compares parameter values for a colloidal system^2^ and for a metallic glass. Considering an Fe-based glass as in the present work, we take *a* to be the Goldschmidt diameter of Fe. It is difficult to estimate *γ′* for a shear band in a MG. For a single shear band operating continuously at a rate corresponding to the overall strain rate on loading, *u*_shear_ is of order 1 µm s^–1^. However, if the operation of a shear band is not continuous, but instead shows serrated (‘stick–slip’) behavior, then *u*_shear_ during the ‘slip’ stages is of order 1 mm s^–1^^51^. Transmission electron microscopy and atomistic simulations suggest an intrinsic shear-band thickness *H* ≈ 20 nm. Considering the zone of enhanced mobility seen in Ref.^44^, we take the upper limit in the current work to be 200 nm. The relative values of *γ′a*^2^/*H* in Table 1 suggest that, especially if the flow is serrated, the shear in the MG is sufficiently intense to induce significant size-segregation.

**Table 1:** Comparison of shear parameters relevant for size segregation in a colloid and in a metallic glass. The parameters representing the colloidal system are from Ref.^2^.

	Colloid	Metallic glass	
		Continuous flow	Serrated flow
Thickness^a^ of flow channel or shear band, *H*	50 µm	20‒200 nm	
Particle or atom diameter^b^, *a*	1.4 µm	252 pm	
Velocity *u*_flow_ or *u*_shear_	250 µm s^‒1^	1 µm s^‒1^	1 mm s^‒1^
Shear rate, *γ′* (s^‒1^)	10	5–50	5 × 10^3^ to 5 × 10^4^
Relative segregation rate, (*γ′⋅a*^2^/*H*)/( *γ′⋅a*^2^/*H*)_colloid_	1	10^‒3^ to 1	1 to 10^3^

^a^In Ref.^2^ the symbol *H* represents the half-thickness, rather than the thickness.

^b^The diameter is for the smaller colloid particles, and for the majority small atom (Fe) in the MG in the current work.

We consider the relative particle sizes for which segregation is seen; in the colloidal system^2^ used to obtain the parameter values in Table 1, the diameters of the large and small particles are in the ratio 2.1. In a simulated granular system also showing size-segregation^47^, the ratio is 1.5. The ratio of the Y and Fe diameters in the present work is 1.44. Comparison of these ratios again suggests that it is reasonable to expect shear-driven size-segregation in the present work. The solid volume fraction (packing efficiency) is lower in colloidal and granular systems than in an atomic condensed phase such as a MG. Yet studies of colloidal and granular systems have extended to fractions as high as 60%, close to the 64% characteristic of dense random packing of an atomic system^2^. According to the mapping for the colloidal system in Ref.^2^, and extrapolating to the relevant large:small radius ratio (i.e. for Y:Fe) and packing efficiency, the present MG is very clearly in the regime in which it is expected that the smaller species (Fe) would segregate toward the center of a flow channel. As noted already, migration to the center of a flow channel implies migration away from the central plane of a shear band. Thus, we expect that, in the present MG, the smaller species should migrate away from the central plane on shear, and that (as, we suggest, is observed) the largest species (Y) should migrate towards, or remain near, the central plane. This direction of migration is also indicated by the results of computer simulation, which showed that the areas with a larger excess volume in a sheared MG are enriched in larger atoms^52^.

The question of how much shear is needed to see significant segregation has been studied for colloid flow^2^. After some time, a steady-state segregation profile is established along the flow channel, reflecting a balance between shear-driven migration and the natural mixing. The transport distance along the channel necessary to achieve the steady-state profile is roughly 1000 × the half-width of the channel. For the case of a shear band of thickness 20‒200 nm, the steady state would be achieved for a shear distance of 10‒100 µm, much longer than reasonable shear distances in the present wear tests. We conclude that the observed segregation does not represent a steady state.

### Possible explanations of the observed effects

The tip is scanned to-and-fro ten times to make the wear track. Given the scan speed and the frequency of oscillatory loading (Fig. 1a), the deformation can be better described as a series of indents than as a plowing action of the tip. Assuming that the indents are uniformly dispersed along the length and across the width of the wear track, they are spaced approximately 50 nm apart. Approximating the crystalline regions as disks 300 nm in diameter (their width in Fig. 2), each one lies under a surface area indented ~ 25 times, and therefore subjected (locally) to 25 cycles of loading and unloading.

We consider the formation of a crystallite of bcc Y solid solution in a shear band in which there is size segregation of the kind depicted in Fig. 4. This crystallite is likely to grow into a plate lying in the plane of maximum shear rate. With successive cycles of loading and unloading, the smaller, faster (Fe, Cr, Mo) metallic atoms are dispersed further into the matrix on either side, leaving the central plane enriched in yttrium. Similar enrichment of a shear band in another RE metal, Tb, was found in a Tb_75_Fe_25_ nanoglass^53^; in this case, however, the system is RE-rich, and the observed enrichment is more easily explained.

**Figure 5:**
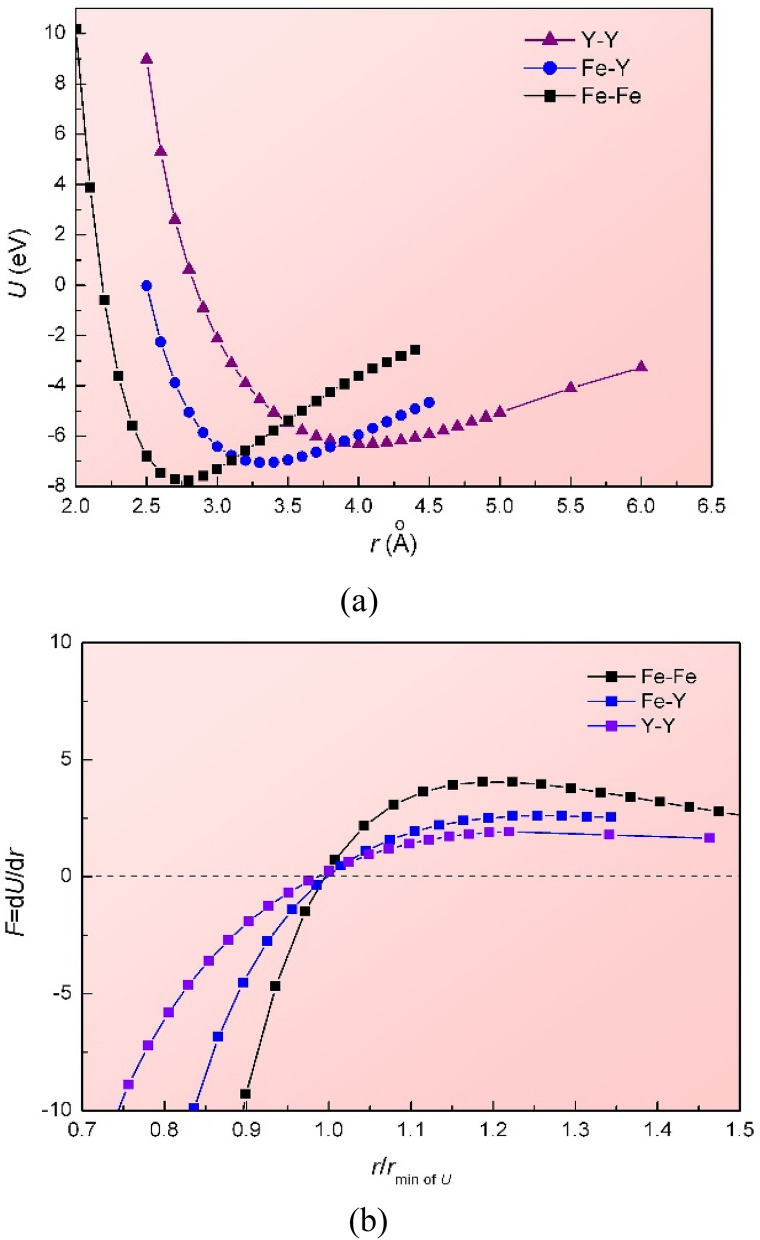
Interatomic interactions of Fe–Fe, Y–Y and Fe–Y atomic pairs by first-principles computer simulation. (**a**) Interatomic potentials (*U*) as a function of absolute distance (*r*), and (**b**) interatomic forces (*F*) as a function of *r* normalized by the value at the energy minimum, for three types of interatomic pair.

Even if the main effect of the shearing is expulsion of smaller atoms from the central plane, the marked concentration of yttrium on that plane implies that the yttrium must have significant mechanical mobility, even when its thermal diffusivity is low. The mechanical mobility can be considered in terms of interatomic potentials calculated using density functional theory (DFT). The interatomic potentials and forces acting between Fe and Y atoms are shown in Fig. 5. The Y–Y and Fe–Y atomic pairs show softer interactions than Fe–Fe. This correlates with the elastic moduli of the elements; the shear modulus *G* is 82 GPa for iron and 25.6 GPa for yttrium; the bulk modulus *B* is 170 GPa for iron and 41.2 GPa for yttrium. The soft potentials are important for shear, rather than diffusion, assisting mechanically driven motion rather than thermally driven motion. Effects of the soft Fe–Y potential may also be seen in the surprisingly high tensile plasticity and low yield stress (~ 100 MPa) of the CuY B2 fully ordered intermetallic compound and several similar (late transition metal)-RE compounds^54^.

**Figure 6:**
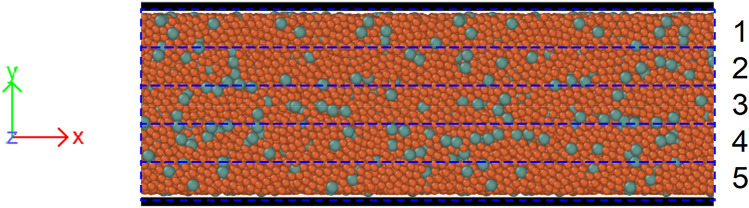
The atomic locations in the central part of the simulation slab after shear deformation. Red atoms—Fe, green—Y. The mole fraction of elements was calculated in plates 1‒5 shown with dashed lines.

We also modeled shear deformation in a Fe_95_Y_5_ model glassy alloy as described in the “Methods” section. Shear deformation right and left for 10 times was applied at the rate of 10^8^ s^‒1^. The initial homogeneous distribution of Fe and Y became inhomogeneous with higher concentration of Y atoms in the center of the channel (Fig. 6). After shear deformation, the Y content in plates 2, 3 and 4 was above 7 at.% while in plates 1 and 5 it was below 5 at.%.

Finally we consider the depth at which the yttrium layers are formed. As shown, for example, in recent work on silicon, which is close to this BMG in compressive strength to *E* ratio^55^, although after mechanical indentation the material is mostly under residual compression, there is an area of biaxial tension under the indent beneath the layer of compressive stresses. In the present study, the finite-element modeling shows strong biaxial tension starting from about 30 nm below the surface (Fig. 1c). Such a stress state could favor aggregation of the large atoms (i.e. Y) and enhance atomic mobility. The likely stress distribution would correspond to the formation of nanoscale bcc Y layers, not on the surface but at a depth of several tens of nanometers.

The oscillating-load mode used for the tests in the present work is significantly more destructive than sliding wear. This is consistent with earlier work showing that low-cycle fatigue severely reduces the flow stress of a BMG sample compared to continuous loading^56^: only 10‒15 loading cycles led to mechanical failure. The strong effect of cyclic loading was attributed to the inhomogeneous distribution of internal stresses and the amplitude of elastic-strain-driven reverse shear on active shear bands upon unloading^57^. That amplitude is much larger in MGs than in crystalline metals, and applies equally in the current work.

## Conclusion

To-and-fro wear of a Fe_48_Cr_15_Mo_14_C_15_B_6_Y_2_ bulk metallic glass using a diamond tip of radius ~ 25 nm induces structural change. Layers of a bcc Y-based solid solution develop directly underneath the wear track, at a depth of 40‒100 nm. These layers are ~ 20 nm thick, and their relative content of metallic elements is Y_84_Fe_8_Mo_5_Cr_3_ (at.%). This remarkable degree of concentration of Y with low thermal diffusivity is attributed to a mechano-chemical pumping action in intensely to-and-fro sheared regions in the MG under the diamond tip. Shear-induced segregation has been studied for particles of different size in colloids and in granular media. We propose that a similar shear-induced segregation of different atomic sizes can occur in the BMG, and we show that the direction of segregation (large atoms, i.e. Y, toward the central plane of a shear band) is consistent with the studies of colloids and granular media. The concentration of yttrium on shear planes is facilitated by the extreme gradients in shear rate characteristic of shear bands in metallic glasses. It mainly reflects expulsion of smaller, more mobile, atomic species (Fe,Cr,Mo) from the central plane, but is also facilitated by the soft Fe-Y interatomic potential that increases the mobility of the relatively slow yttrium. The discovery of this mechano-chemical pump effect is of fundamental interest, but also merits investigation for potential applications.

## Methods

### Sample preparation

An ingot of the Fe_48_Cr_15_Mo_14_C_15_B_6_Y_2_ alloy was prepared by arc-melting the mixtures of pure metals and metalloids (above 99.9 mass.% purity) under an argon atmosphere. A BMG rod, 2 mm in diameter was prepared by using induction melting and subsequent copper-mold casting.

### Plastic deformation

Atomic force microscopy (AFM) with a single-crystal diamond tip mounted on the cantilever (AFM Probe D300, SCDprobes) was used to obtain the topography profiles in tapping mode. The wear tests were performed with constant load of 1 μN or with load oscillating around 1 μN (from 0 to 2 μN) at the frequency of 13 Hz (Fig. 1a). The cantilevers have a typical spring constant of 40 N m^‒1^, measured from the change in resonant frequency of the fundamental mode of vibration^57^. For each AFM session, a new cantilever was taken. Each fresh cantilever has a rounded tip of radius ~ 25 nm. The wear tests were performed with a tip scan velocity of 1 µm s^‒1^^44^.

The reduced or indentation modulus *E*_r_ is determined on unloading, and by using the Hertzian elastic solution to fit the shape of the load–displacement curve up to the first pop-in:

1 $$\frac{1}{{E_{{\text{r}}} }} = \frac{{\left( {1 - \nu _{{\text{i}}}^{2} } \right)}}{{E_{{\text{i}}} }} + \frac{{\left( {1 - \nu ^{2} } \right)}}{E},$$

where *E* and ν are Young’s modulus and Poisson’s ratio of the studied metallic glass, respectively, while the corresponding values for the diamond indenter tip are *E*_i_ = 1141 GPa and *ν*_i_ = 0.07. Vickers microhardness measurement was conducted at 4.9 N load.

### Finite-element modeling

The stress distribution inside the BMG sample in three dimensions loaded with a diamond tip 50 nm in diameter was modeled with the finite-element modeling (FEM) software DEFORM (see details in Supplementary Information S2.3).

### X-ray photoemission spectroscopy

X-ray photoemission spectroscopy (XPS) studies of the sample surface were performed using the electron–ion spectroscopy module based on Nanofab 25 (NT-MDT) platform in an oil-free ultrahigh vacuum of 10^–6^ Pa. The X-ray source SPECS XR 50 with Mg Kα radiation (1253.6 eV photon energy) without a monochromator was used. The spectra were recorded with an electrostatic hemispherical energy analyzer SPECS Phoibos 225. The energy resolution based on the full width at half maximum of the Ag3d5/2 line (peak) was 0.78 eV. The energy positions of the spectra were calibrated with reference to the Cu2p3/2 (binding energy (BE) of 932.62 eV), Ag3d5/2 (BE of 368.21 eV) and Au4f7/2 BE of 83.95 eV) peaks. The survey spectra scans were recorded at a pass energy of 80 eV. The detailed scans of strong lines (peaks) were recorded at a pass energy of 20 eV as wide as needed to encompass the peak(s) of interest.

An ion source SPECSIQE 12/38 fed with 99.9995% pure Ar was used to clean organics from the samples at an incidence angle of 60° to the surface normal with an energy of 500 eV and an ion current of 100 A. The scanned area was 2.8 × 4.0 mm^2^. The sputtering time was 10 min. The chemical shift energy *ES* = *zES*_1_ depends almost linearly on the oxidation rate *z* of the element; here *ES*_1_ is the chemical shift energy per unit of oxidation rate. The values of chemical shifts for oxides and carbides were used to find *ES*_1_ of the O1s, C1s, Fe2p, Mo3d, Cr2p and Y3p lines (peaks)^58^.

### Transmission electron microscopy

Transmission electron microscopy (TEM) observation of the samples was carried out using a JEOL JEM2010F and a FEI Tecnai Osiris microscope with field-emission gun and TEM/STEM facility operated at 200 keV, the latter equipped with a Super-X windowless EDX detector for high-resolution elemental mapping. The EDX experiments were performed in scanning TEM mode using a high-angle annular dark-field (HAADF) detector and a probe diameter of ~ 0.2 nm. SAED patterns and associated images were acquired with a Gatan US1000 charge-coupled device camera. The SAED aperture was ~ 200 nm, and the camera length was set to cover the high-*q* range up to 12.2 Å^−1^; the acquisition time was 1 s.

A BMG sample was treated to have five parallel wear tracks. This nano-scratched region was then located by SEM, and a thin layer of platinum (200 nm) was deposited over across the wear tracks using the electron beam (2 nA, 5 kV). A cross-sectional specimen with a final thickness of 50–100 nm was then prepared by FIB milling using a Helios Nanolab FIB/SEM (Thermo Fisher Scientific). This specimen was transferred from the sample to a standard grid for TEM. A standard in-situ procedure was adopted for lift-out. Initial set-up began with deposition of platinum (12 × 2 × 2.5) μm^3^ using the Ga^+^ beam, followed by coarse milling steps using a 30 kV/9 nA beam to remove most of the material. The sample was further thinned to a thickness of < 1.0 μm using a 30 kV/1.5 nA beam and then undercut in preparation for release. Lift-out was achieved by fixing the specimen to an Omni probe needle using platinum, releasing with a FIB cut, and then lifting out with the stage at 0° tilt. Once clear of the bulk material, the specimen was fixed on the standard Omni grid. Further thinning was performed at 30 kV/300‒100 pA, until the platinum appeared transparent in a 5 kV electron-beam image, indicating a thickness of < 150 nm. Final polishing was at a reduced voltage of 2 kV to further thin the lamella to final electron transparency at 3 kV electron energy. This procedure minimizes the amount of damaged material and reduces the levels of implanted gallium in the thin foil.

Scanning electron diffraction (SED) experiments were conducted in JEOL ARM300F double corrected (S)TEM at 300 kV. A highly parallel beam with around 0.6 mrad convergence angle was used. It corresponds to a 5 nm beam size in real space. The diffraction patterns were recorded with a step of 7.1 nm in 256 × 256 array by the use of the Merlin/medipix direct electron detector system. The scattering vector (*q*) range was set to approximately 10 Å^‒1^. The data processing was performed using the Pyxem package^59^.

### First-principles calculations

First-principles calculations of bcc Y solid solution based on density-functional theory^60,61^ were performed using the Vienna Ab initio Simulation Package (VASP)^62,63^, with the Perdew–Burke–Ernzerhof (PBE) exchange–correlation functional^64^ and projector augmented-wave (PAW) pseudopotentials^57,65^. The cut-off for the kinetic energy was set to 270 eV and the *k*-point sampling was a 12 × 12 × 12 Monkhorst–Pack grid^66^. For each structure, the cell parameters and atomic positions were relaxed until all forces are smaller than 0.01 eV Å^‒1^.

### Classical molecular dynamics simulations

Shear deformation of Fe_95_Y_5_ glassy alloy was modeled by classical molecular-dynamics simulation using the Large-scale Atomic/Molecular Massively Parallel Simulator (LAMMPS) software package with GPU acceleration^67,68^. The interatomic interactions were described by a bond-order potential as introduced by Mock and Albe^69^. The sample, made of 275,169 atoms is first melted at 2000 K for 200 ps, then cooled down to 1500 K at which ten cycles of shear loading and unloading are performed at 10^8^ s^‒1^. Open boundaries are applied in the *x* and *y* directions while periodic boundary conditions are applied along the *z* axis (see Fig. 6). Shear is applied along the *x* axis. The time step was set to 2 fs. The temperature and pressure were controlled using Nosé–Hoover style non-Hamiltonian equations of motion^70,71^.

## Supplementary Information

Supplementary Video 1.

Supplementary Information.

## Data Availability

The data that support the findings of this study are available from the corresponding author on request.
