# Supplementary material for: Shear-induced chemical segregation in a Fe-based bulk metallic glass at room temperature
Source: Sci Rep. 2021 Jul 1;11:13650. doi: 10.1038/s41598-021-92907-4 (PMC8249454; doi:10.1038/s41598-021-92907-4)
Supplement: Supplementary file 2 — Supplementary Information. [file 41598_2021_92907_MOESM2_ESM.pdf]

Supplementary Information

Shear-induced chemical segregation in a Fe-based bulk metallic glass at room temperature

D. V. Louzguine-Luzgin, A. S. Trifonov, Yu. P. Ivanov, A. K. A. Lu, A. V. Lubenchenko, A. L. Greer

S1. Structural characterization of as-prepared samples

The as-solidified $Fe_{48}Cr_{15}Mo_{14}C_{15}B_6Y_2$ (at.%) alloy rod of 2 mm diameter was found to be glassy by using conventional X-ray diffractometry and transmission electron microscopy (Fig. S1).

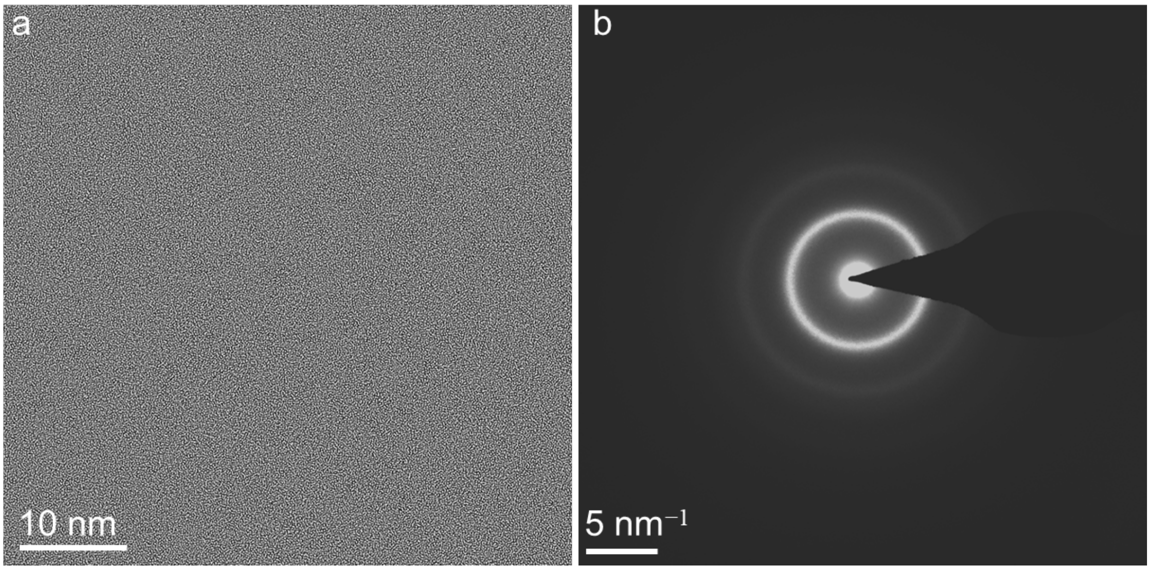

Fig. S1. High-resolution TEM image (a) and SAED pattern (b) of the as-solidified $Fe_{48}Cr_{15}Mo_{14}C_{15}B_6Y_2$ sample.

The obtained XPS spectra are shown in Fig. S2. Circles show the recorded detailed spectra where the solid lines are calculated using the method proposed earlier [1], while fitted areas represent separate calculated peaks. Carbon can hardly be separated from surface-absorbed carbon, though traces of carbides were formed. The boron content was too low to be detected.

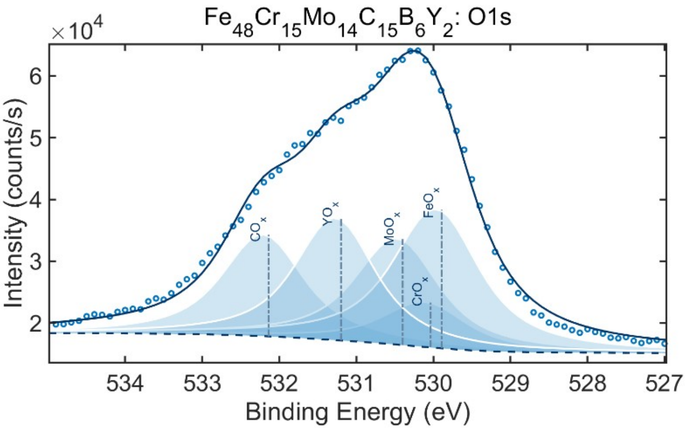
Fe48Cr15Mo14C15B6Y2: O1s
×10^4
Intensity (counts/s)
Binding Energy (eV)
COx
YOx
MoOx
FeOx
CrOx
534 533 532 531 530 529 528 527
2 3 4 5 6
(a)

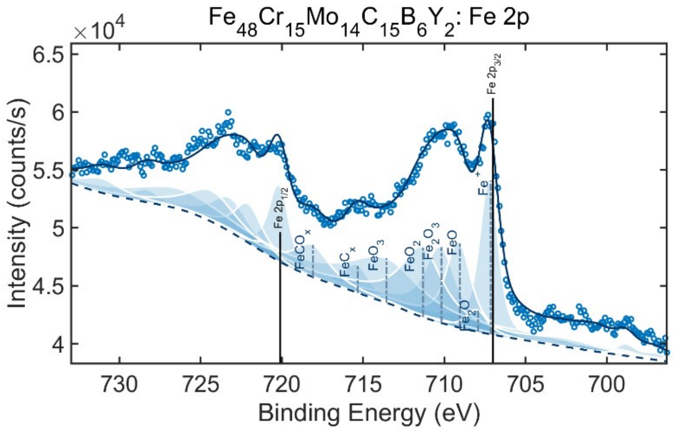
Fe48Cr15Mo14C15B6Y2: Fe 2p
×10^4
Intensity (counts/s)
Binding Energy (eV)
Fe 2p3/2
Fe 2p1/2
FeCOx
FeCx
FeO3
FeO2
Fe2O3
FeO
Fe2O
Fe+
730 725 720 715 710 705 700
4 4.5 5 5.5 6 6.5
(b)

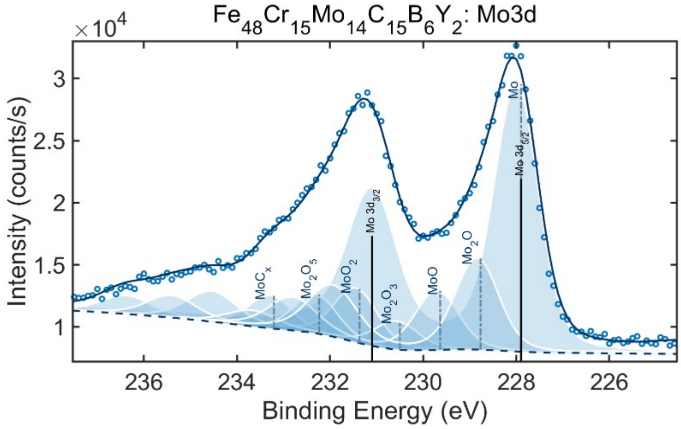
Fe48Cr15Mo14C15B6Y2: Mo3d
×10^4
Intensity (counts/s)
Binding Energy (eV)
Mo
Mo 3d5/2
Mo 3d3/2
MoCx
Mo2O5
MoO2
Mo2O3
MoO
Mo2O
236 234 232 230 228 226
1 1.5 2 2.5 3
(c)

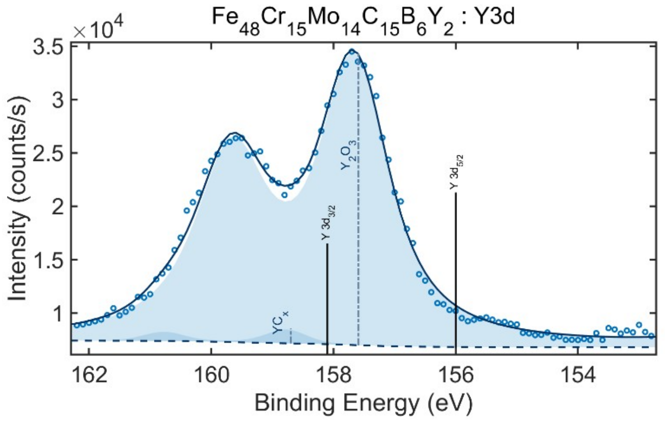
Fe48Cr15Mo14C15B6Y2 : Y3d
×10^4
Intensity (counts/s)
Binding Energy (eV)
Y2O3
Y 3d3/2
Y 3d5/2
YCx
162 160 158 156 154
1 1.5 2 2.5 3 3.5
(d)

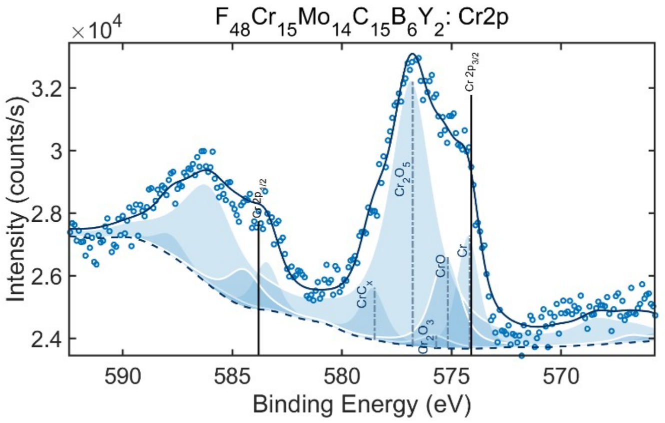

(e)

Fig. S2. XPS spectra. Lines (a) O 1s, b) Fe 2p, (c) Mo 3d, (d) Y 3p and (e) Cr 2p. Dots: experimental data, solid lines: calculation. Filled areas: separate calculated peaks.

Non-destructive chemical and phase depth-profiling of nano-sized films in this investigation was carried out using the standard method [1]. This method enables determination of the depth profiles with a sub-monolayer accuracy from XPS data. The determined chemical composition of the 1.7 nm thick oxide layer is shown in Table S1. All four constituent metals were found to be present in the form of surface oxides.

Table S1. Layer chemical composition, thickness and volume fraction of the substances.

Layer	*h*, nm	Volume fractions of the constituents
Oxide	1.7	0.48 FeO_x + 0.10 MoO_2+ 0.16 Y_2O_3 + 0.24 CrO_x
Substrate	infinite	0.59 Fe + 0.30 Mo + 0.11 Cr

S2. Scratch-wear tests

S2.1. Diamond tips

An example of the diamond tips used for the scratch-wear tests is shown in Fig. S3.

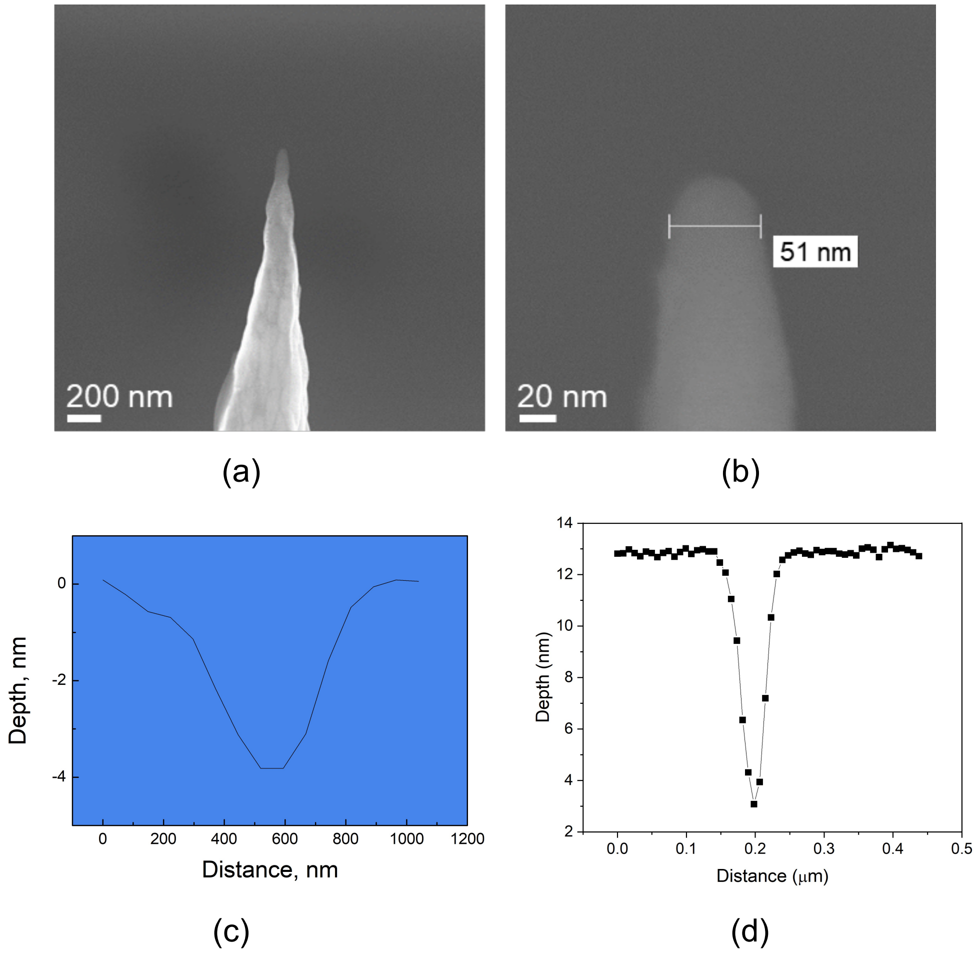

Fig. S3. Diamond cantilever tip used for the scratch test: (a) at a lower and (b) at a higher magnification; scanning electron microscopy. Wear profile (c) obtained at oscillating load of 0-2 μN and wear profile obtained after 100 wear runs at constant (not oscillating) load of 1 μN (d) .

S2.2. Normalized pressure and temperature calculations

In order to check whether temperature rise is likely during the wear, we consider the wear maps [2]. The normalized sliding velocity (ν') is given by:

$$\upsilon' = \frac{\upsilon r}{\alpha} \qquad (1)$$

where v is the sliding velocity used in the test (1 μm s^{-1}), α is the thermal diffusivity of the BMG (~2 mm^2 s^{-1}) [3] and r is the radius of the circular nominal contact area (~25 nm). The resulting v' value of ~$9{\cdot}10^{-9}$ lies firmly in the regime of mild wear.

The normalized pressure P' is given by:

$$P' = F/(A \cdot H_v) \qquad (2)$$

where F is the applied normal load, A is the nominal contact area of the wearing surface, (2×10^3 nm^2 estimated from the scratch width), and H_V is the room-temperature Vickers hardness (12 GPa]) of the BMG sample. The resulting normalized pressure at 1 μN average load is 5×10^{-2} which corresponds to delamination wear conditions.

The contact surface temperature (T_s) is given by:

$$T_s = T_0 + \frac{\mu T^* \beta}{2+\beta\sqrt{\pi v'/8}} P' v' \qquad (3)$$

where μ is the coefficient of friction, β is close to one, T^* is the equivalent temperature $T^*=a{\cdot}H_v/K_m$, and K_m is the thermal conductivity (~12 W m^{-1} K^{-1} [4]). According to the calculations, the normalized pressure is 10^{-2} and the normalized speed 5×10^{-8}. Substituting values into Eq. (2), the temperature rise is found to be negligible (~10^{-7} K).

S2.3. *Finite-element modeling*

The stress distribution inside the BMG sample in three dimensions loaded with a diamond tip 50 nm in diameter was modeled with the finite-element modeling (FEM) software DEFORM. The specimen was divided into 30,000 tetrahedral elements. The elastic properties of the BMG were taken to be: E, 210 GPa; G, 80 GPa; B, 200 GPa; HV, 12 GPa; Poisson ratio, 0.325. These values, together and the stress-strain curves [5,6], were used for the FEM calculations together with elastic properties of diamond.

S3. Comparison with a typical Zr-based BMG

No strain hardening or increase in the Young modulus E is found for a $Zr_{62}Cu_{22}Fe_5Al_{10}$ BMG also tested in oscillating mode as Fe-based BMG (Fig. S4). Fig. S5 shows the results of STEM observation of a nanoscale scratch formed in the $Zr_{62}Cu_{22}Fe_5Al_{10}$ BMG.

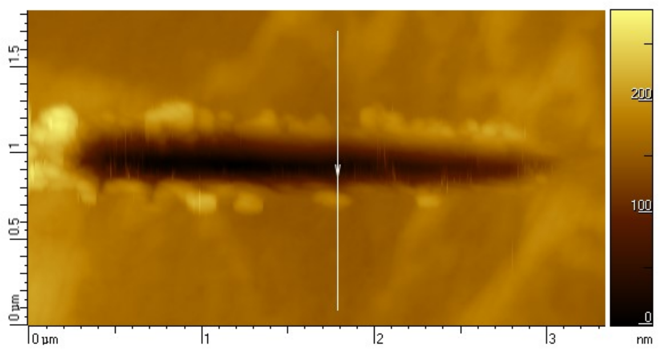

(a)

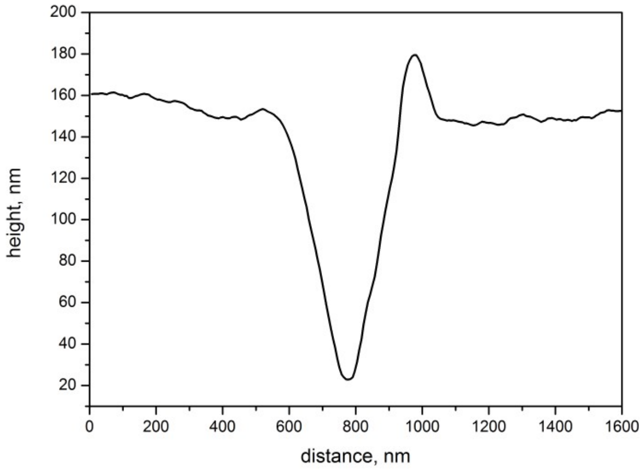

(b)

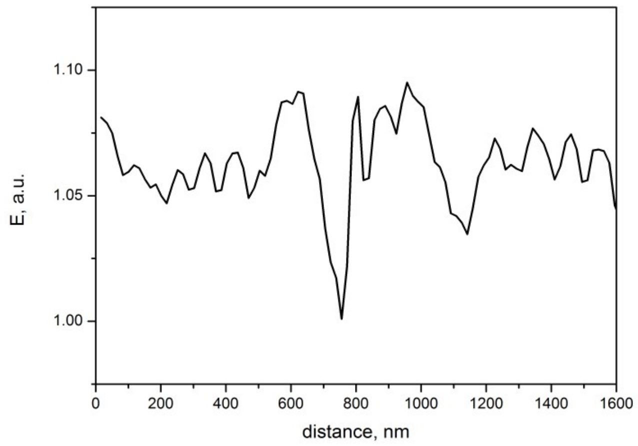

(c)

Fig. S4. (a) Surface topology, (b) Young's modulus distribution on the surface and in the scratch and (c) profile of the Young's modulus of the $Zr_{62}Cu_{22}Fe_{5}Al_{10}$ BMG. Ten scratch runs with load oscillating between 0 and 200 nN.

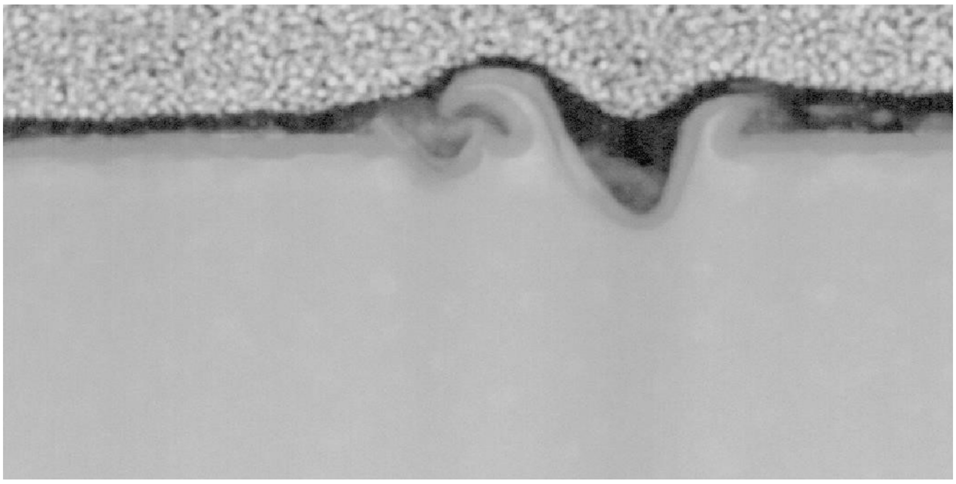

Fig. S5. STEM observation of a nanoscale scratch formed in the $Zr_{62}Cu_{22}Fe_5Al_{10}$ BMG. No crystallization is found. The profile is evidently very different from that shown in the main text (Fig. 2 and Fig. 3) for the Fe-based BMG in the present work.

S4. An atomic cell simulated using first-principles calculations

Ab-initio simulation of BCC Y containing 128 atoms (4 Cr, 10 Fe, 6 Mo and 108 Y) confirmed that the lattice parameter of such a solution containing 16 at.% of the solute elements is 380 pm which is quite close to the experimentally measured value. The resulting atomic cell is shown in Fig. S6.

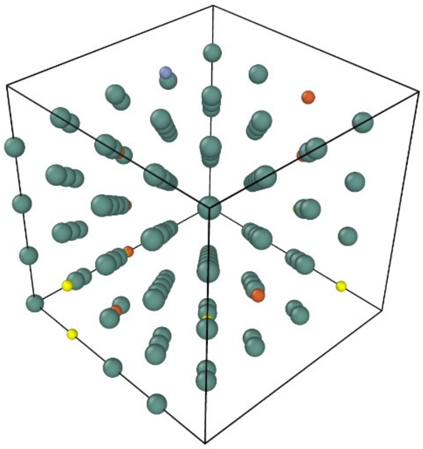

Fig. S6. The calculated atomic cell of Y (turquoise) containing 8 at.% Fe (red), 3 at.% Cr (blue) and 5 at.% Mo (yellow).

We modeled shear deformation in a $Fe_{95}Y_5$ model glassy alloy as described in the Methods

section. Corresponding video file is also attached to the manuscript.

References

1. A.V. Lubenchenko, A.A. Batrakov, A.B. Pavolotsky, O.I. Lubenchenko, D.A. Ivanov, XPS study of multilayer multicomponent films, Appl. Surf. Sci. A 427, (2018) 711–721.
2. M.F. Ashby, S.C. Lim, Wear-mechanism maps, Scripta Metall. Mater. 24 (1990) 805–810
3. C.L. Choy, W.P. Leung, Y.K. Ng, Thermal conductivity of metallic glasses, J. Appl. Phys. 66 (1989) 5335–5339.
4. R. Nowosielski, A. Januszka, R. Babilas, Thermal properties of Fe-based bulk metallic glasses, J. Achiev. Mater. Manufact. Eng. 55 (2012) 349–354.
5. X.J. Gu, S. J. Poon, G. J. Shiflet, Mechanical properties of iron-based bulk metallic glasses, J. Mater. Res. 22 (2007) 344–351.
6. C. Suryanarayana, A. Inoue, Iron-based bulk metallic glasses, Int. Mater. Rev. 58 (2013) 131–166.
